# Supplementary material for: Identification of photoperiod-induced specific miRNAs in the adrenal glands of Sunite sheep (Ovis aries)
Source: Front Vet Sci. 2022 Jul 22;9:888207. doi: 10.3389/fvets.2022.888207 (PMC9354845; doi:10.3389/fvets.2022.888207)
Supplement: Supplementary file 1 [file Data_Sheet_1.ZIP › Supplementary file/miRDeep2 file.zip!/miRDeep2 file/Novel_Structure/SP42A1/Novel_316.pdf]

Provisional ID : NC_019464.2_10993
Score total : 2
Score for star read(s) : -1.3
Score for read counts : 0
Score for mfe : 1.7
Score for randfold : 1.6
Score for cons. seed :
Total read count : 63
Mature read count : 63
Loop read count : 0
Star read count : 0

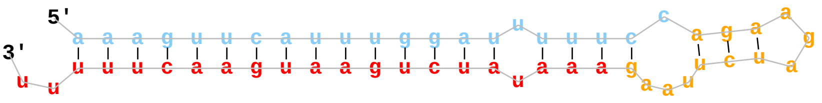

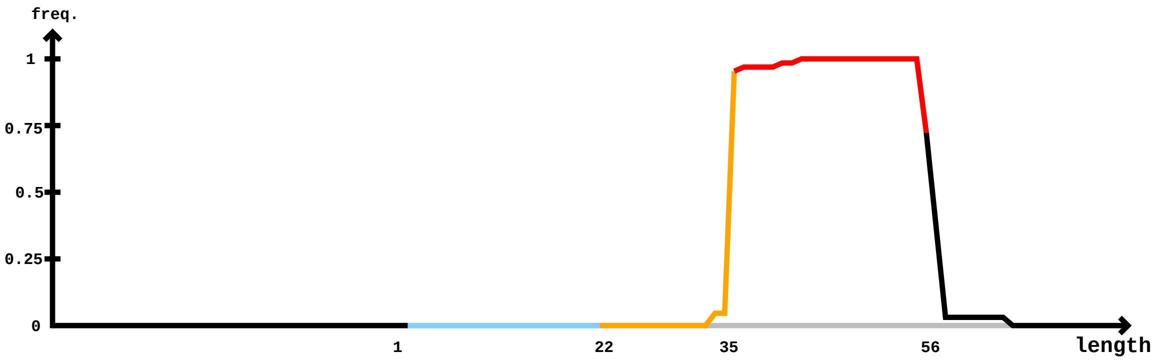

Star Mature

```
5'- ugucauaaaaggguaaauacuguugaguuggucaaaaaguucauuuggauuuuuccagaagaucuuaagaaauaucugaaugaacuuuuuggccaacccgauuuauaauu   -3'    exp
    .....................((((.((((((((((((((((((((((((.((((.(((...)))...)))).)))))))))))))))))))))))).)))).........   reads    mm    sample
    ...................................................................agaaaAaucugaaugaacuuuu.....................   1        1     seq
    ...................................................................agaaaAaucugaaugaacuuuuu....................   2        1     seq
    .....................................................................aaaAaucugaaugaacuuuu.....................   17       1     seq
    .....................................................................aaaAaucugaaugaacuuuuu....................   42       1     seq
    ......................................................................aauaucugaaugaacuuuuu....................   1        0     seq
    ..........................................................................ucugaaugaacuuuuuggccaacA............   1        1     seq
    ............................................................................ugaaugaacuuuuuggccCacc............   1        1     seq
```
